# Case report: Challenges and implications of conduction system pacing in pediatrics: Case series

**DOI:** 10.3389/fped.2023.1160335

**Published:** 2023-05-04

**Authors:** Ruy Gonzalo Ploneda Valencia, Moisés Levinstein Jacinto, Carmen A. Sánchez Contreras, Gustavo Ruiz González, Diego Neach de la Vega, Manlio F. Márquez Murillo, Santiago Nava

**Affiliations:** Department of Electrophysiology, Ignacio Chávez National Institute of Cardiology, Mexico City, Mexico

**Keywords:** children, pacemaker, physiological stimulation, His bundle pacing, left bundle branch pacing, congenital heart block

## Abstract

Cardiac electrical stimulation in children usually is needed in the setting of complete congenital atrioventricular block, atrioventricular block after heart surgery, and bradycardia associated with some specific channelopathies. In cases of atrioventricular block, the high percentage of ventricular stimulation raises concern on the deleterious effects of chronic stimulation of the right ventricle. In recent years, physiologic stimulation has developed as a valid approach for adult patients and a great interest has risen in offering conduction system pacing also to the pediatric population. We present three pediatric cases of stimulation of the conduction system (His bundle or left bundle branch), in order to show the intrinsic particularities and challenges implied in these new techniques.

## Introduction

Since the first pacemaker (PM) implant performed in 1958 by Senning and Elmqvist (1, 2), research on stimulation techniques have progressed markedly. Direct stimulation of the His bundle in humans was described in the year 2000 (3) and the first case of selective stimulation of the left bundle branch in 2017 (4). Techniques were developed for the adult population with heart failure, which required cardiac resynchronization. In pediatric population, we face different scenarios in which a patient may require pacemakers. Considering that the pediatric population usually requires high percentages of ventricular stimulation, close to 100% (5), and the known deleterious effects of chronic cardiac stimulation from the apex of the right ventricle (6, 7), a great interest has risen in attempting physiological stimulation in this age group.

In the case of implantable cardioverter defibrillators (ICDs), the pediatric recommendations have been limited by the lack of randomized controlled trials and small patient numbers, and the implant indications are extrapolated from adult data, which is based on specific diagnosis as the defined cause or presumed risk factor for sudden cardiac arrest (SCA), such as ischemia, cardiomyopathy, or genetic cardiovascular disease. Almost half of the cases of SCA in the pediatric population remain undefined, and, in the case of channelopathies, SCA is often the initial symptom of the disease. Recently, the 2021 PACES Expert consensus statement published guidelines for implantable electronic device in pediatrics, including ICDs, where they make the observation that are still extensive “gaps” in current ICD recommendations, irrespective of age, for many of the diseases associated with SCA in pediatrics, and the recommendations are based on limited clinical data or expert opinion and consensus (8).

We report three cases of physiological stimulation, Hisian (*n* = 1) and left bundle branch (*n* = 2), in Mexican children with different clinical conditions, to show the intrinsic particularities and challenges implied in these techniques.

## Case reports

### Case 1

**Figure 1:**
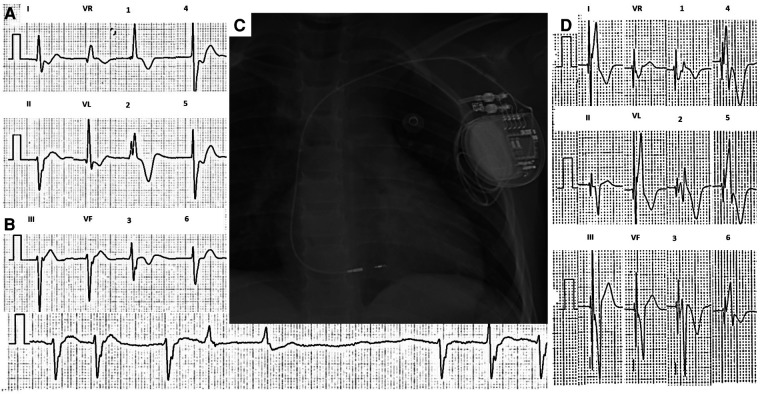
Twelve-lead ECG of case 1 taken at 10 mm/mV and 25 mm/s. (**A**) Sinus arrest and AV junctional rhythm at a rate of 42 beats per minute with a QRS of 140 ms and bifascicular block, with no evidence of atrial activity. (**B**) 2.3 s pause during sinus arrest. (**C**) Post-procedure chest x-ray. (**D**) 12-lead ECG with nonselective left bundle branch stimulation. ECG, electrocardiogram.

A 5-year-old girl weighing 16.5 kg presented with a 3-month history of syncope. Electrocardiogram (ECG) documented sinus arrest, with pauses of up to 2.3 s and junctional0 rhythm at 37 bpm with hemodynamic compromise requiring temporary PM placement (Figures 1A, B). There was no significant family history. Echocardiogram was normal with ventricular septal thickness of 6.4 mm.

Electrophysiology study showed the absence of electrical activity in both atria, with inability to capture in various locations at maximum voltage (Supplementary Material S1A); with very sporadic atrial depolarizations, atrium-his (AH) and his-ventricle (HV) intervals were prolonged (Supplementary Material S1B). Due to the possibility of coexistence between diffuse cardiac electrical disease and ventricular arrhythmias (9), a ventricular tachycardia induction protocol was performed, which was negative. She underwent single-chamber PM implantation with stimulation of the His bundle using the SelectSecure MRI SureScan 3830 electrode and the C315 sheath (Medtronic, Minneapolis, MN, United States). The lead was implanted 1 cm below the anatomical region of the His bundle, at the left bundle branch. Stimulation at this location managed to capture the left bundle branch and generate a QRS of 115 ms, the threshold was 0.7 V/1.0 ms and unipolar impedance was 855 Ω. Contrast injection through the sheath allowed evidence of adequate penetration of the electrode in the interventricular septum (Supplementary Material S1C,D). The final ECG showed a narrower QRS with a right bundle branch image compared to the baseline QRS (from 140 to 115 ms) with the same axis as the intrinsic rhythm (Figure 1D). Total fluoroscopy time was 8.6 min and 89.1 mGy. During follow-up, an SCN5A mutation was documented and has been fully reported by Villarreal-Molina et al. (9).

### Case 2

A 12-year-old boy weighing 39.5 kg with Holt–Oram syndrome and a persistent left superior vena cava was sent to our service due to complete atrioventricular (AV) block, significant decrease in functional capacity, and chronotropic incompetence during the stress test.

The initial ECG showed a complete AV block with normal axes, QRS of 80 ms, and a mean ventricular rate of 40 bpm (Figure 3A).

Dual-chamber endocardial PM with stimulation of the His bundle was attempted. Before starting the procedure, a venography was performed, demonstrating the absence of the brachiocephalic vein and a direct connection of the left venous system to the coronary sinus (Figure 3C). Therefore, the right axillary vein approach was used.

**Figure 2:**
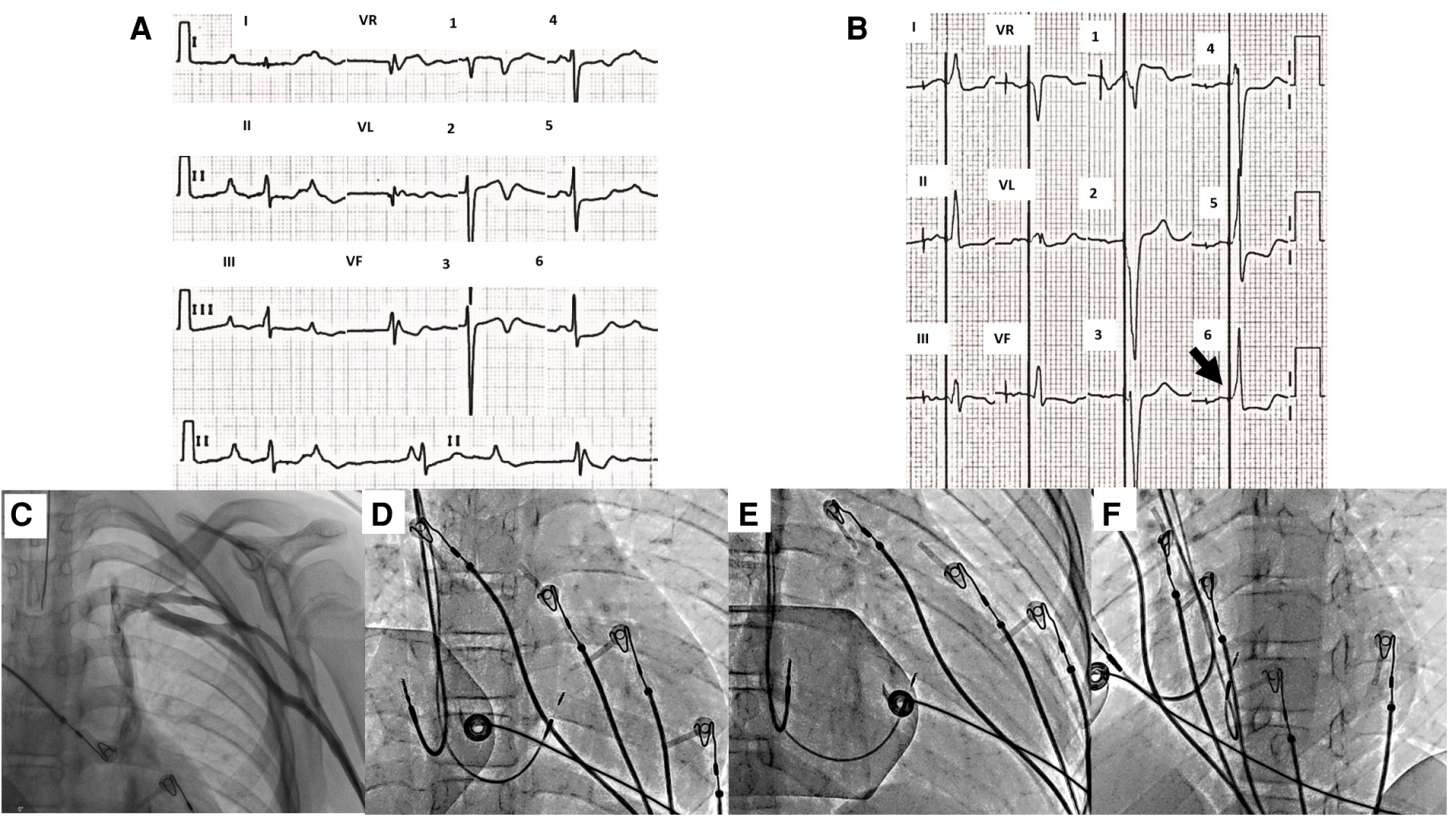
(**A**) 12-lead ECG of case 2 taken at 10 mm/mV and 25 mm/s. Complete AV block with normal axes. The mean atrial rate is 70 bpm, and the mean ventricular rate is 40 bpm. (**B**) 12-lead ECG taken at 10 mm/mV and 25 mm/s. Unipolar dual-chamber pacing with heart rate at 63 bpm, normal P-wave, and QRS axes. After ventricular stimulation, an immediate onset of the QRS with pseudodelta (black arrow) is observed, which was measured at 90 ms at II, III, aVF, and V6. (**C**) Venography shows an absent connection of the brachiocephalic vein and a direct connection of the left venous system to the coronary sinus. The final position of the leads in (**D**) anteroposterior view, (**E**) right oblique 30°, and (**F**) left oblique 30° are shown. ECG, electrocardiogram; AV, atrioventricular.

Ultrasound-guided puncture of the right axillary vein was performed on two occasions and two 7 Fr introducers were placed; through the first, the atrial electrode was advanced to the appendage of the right atrium, and adequate capture was evidenced. Through the SelectSite C304-His deflectable sheath (Medtronic, Minneapolis, MN, United States), the SelectSecure MRI SureScan 3830 electrode was advanced to the anatomical region of His, documenting a supra-Hisian block with an HV interval of 33 ms (Supplementary Material S2A). The electrode was implanted in that area, and a fast potential was observed, obtaining nonselective capture with a threshold of 1.2 V/1 ms and unipolar impedance of 1,270 Ω (Supplementary Material S2B). The final ECG showed a QRS of 90 ms when measured in II, III, aVF, and V6, with a pseudodelta wave and a normal axis (Figure 2B). Total fluoroscopy time was 20.2 min and 1,103 mGy. One month after the implant, during follow-up, the threshold improved to 0.9 V/1 ms, and the unipolar impedance to 738 Ω. At follow-up, the patient and the family reported improvement of his functional class (NYHA class I).

### Case 3

A 5-year-old girl weighing 13.5 kg with Down syndrome and a history of congenital heart disease-type perimembranous ventricular septal defect and surgical closure with bovine pericardial patch developed postsurgical complete AV block; therefore, a permanent dual-chamber epicardial PM was implanted in 2017. She was referred to our institute due to functional class deterioration. Intraventricular and interventricular dyssynchrony were evidenced by echocardiography, with significant dilation of the left ventricle (*Z*-score +3.59) and left ventricular systolic and diastolic dysfunction [left ventricular eyection fraction (LVEF) 15%]; there was also a significant tricuspid regurgitation. The thickness of the interventricular septum was measured at 5.4 mm. After ruling out residual defects, PM-induced cardiomyopathy was concluded as etiology of the heart failure.

**Figure 3:**
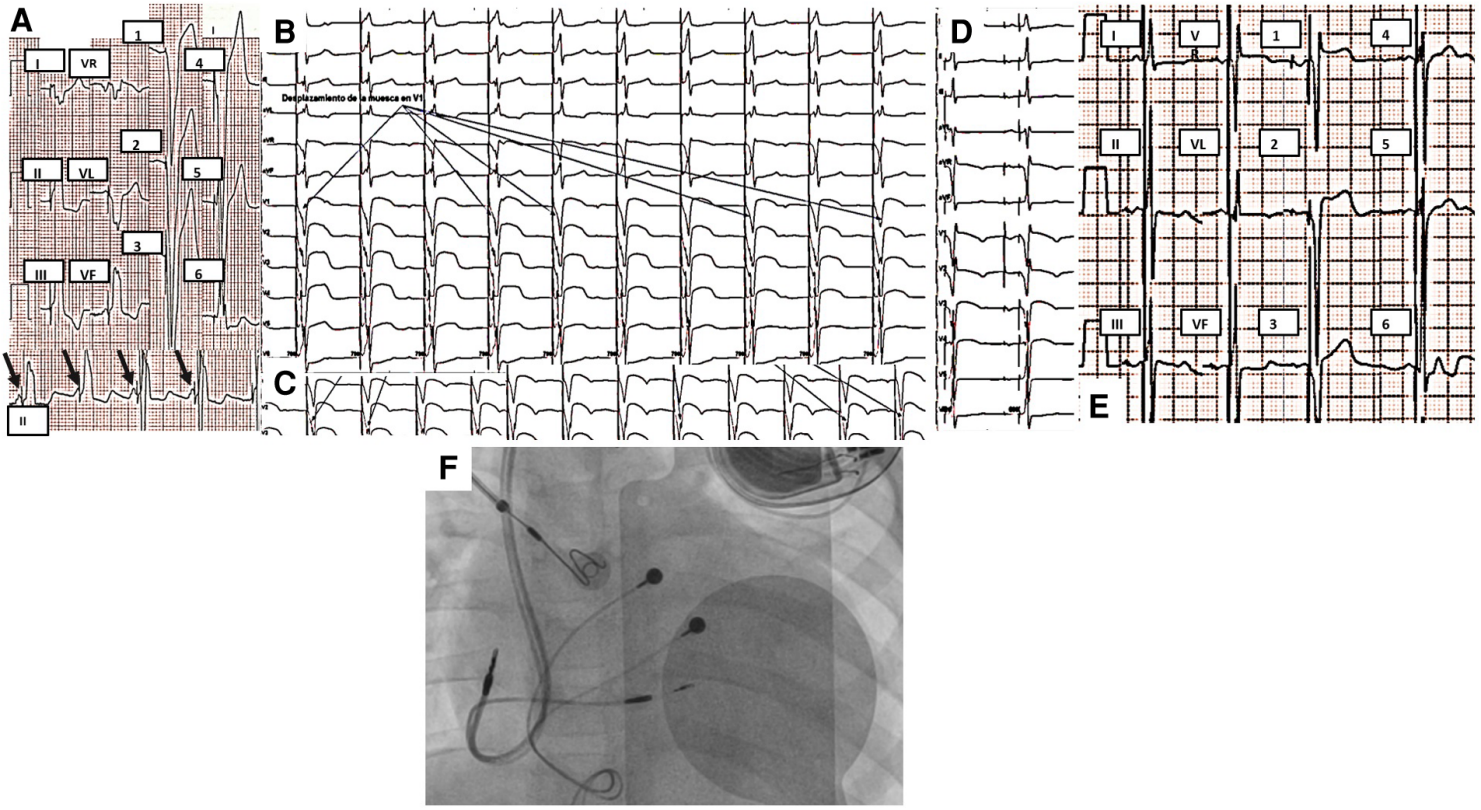
(**A**) Initial 12-lead ECG of case 3 taken at 10 mm/mV and 25 mm/s. PM rhythm with single-chamber pacing. AV dissociation (black arrows point to *P* waves) with a mean atrial rate of 90 bpm, QRS of 160 ms with inferior and right axis, and late transition. (**B**) QRS notch progression during the first three turns observed in multiple surface ECG leads (arrows point to V1) during advancement of the ventricular electrode through the interventricular septum. (**C**) QRS notch progression during the last two turns was observed in V2 during the advancement of the ventricular electrode through the interventricular septum. (**D**) Dual-chamber pacing in the final position of the ventricular electrode with right bundle branch image. (**E**) Selective stimulation of the left bundle branch of His. Final ECG with normal P-wave and QRS axes, 80 ms final QRS with right bundle branch image, and late transition. (**F**) Final position of pacemaker, atrial and ventricular endocardial leads. As it can be seen, the epicardial leads were placed in an unusual position, near the AV junction. ECG, electrocardiogram; AV, atrioventricular.

The admission ECG showed PM rhythm with single-chamber stimulation and atrioventricular dissociation (Figure 3A). The chest radiography showed epicardial leads position near the AV junction.

It was decided to implant a permanent dual-chamber endocardial pacemaker with selective stimulation of the left bundle branch, considering the congenital heart disease, the anatomy of the conduction system, and the surgical correction. Through the left axillary vein, an atrial electrode was placed in the right atrial appendage, where adequate capture was evidenced. Using the C315 sheath, the SelectSecure MRI SureScan 3830 electrode was implanted guided by electrograms. After three turns, it was possible to show left bundle branch potential and a notch in the descending limb of the S wave in V1 (Supplementary Material S3). Two more turns were given to the electrode without presenting ventricular extrasystoles with right bundle branch morphology and observing notch displacement in V1 and V2 to the right, finally showing in V1 and V2 with r′ (Figures 3B, C); the position was confirmed using contrast. Tests performed showed a capture threshold of 1.1 V/1 ms and unipolar impedance of 756 Ω. The final ECG showed a narrow QRS (80 ms), with normal axes and a right bundle branch image in V1 (Figure 3E). Total fluoroscopy time was 31.9 min and 1,579 mGy. An echocardiogram was performed 24 h after PM implantation with left bundle branch stimulation. It demonstrated interventricular synchrony, amelioration of LV dilation (*Z*-Score +2.19), and a significant improvement of left ventricular systolic function as LVEF increased to 31.8%.

## Discussion

The technique and criteria for stimulation of the His bundle and the left bundle branch have been recently described by Vijayaraman and Ponnusamy (10, 11). Physiological stimulation in pediatrics is a technical challenge due to the variety of anatomical conditions and electrophysiological properties that can occur in this population (12, 13). There are few reports in the literature of physiological stimulation in the pediatric population, none of them involving patients with diffuse electrical disease and focusing mainly on patients with postsurgical complete AV block (14–20).

### First challenge: anatomy of pediatrics subjects

Anatomical differences in the pediatric population early in life have encouraged the preferential use of epicardial pacing in very young patients. In our institute, endocardial pacemakers have been placed in patients from 6 months of age and weighing 4.4 kg; however, these cases are the exception and not the rule (21). The epicardial stimulation reverses physiological ventricular activation, increasing transmural dispersion of repolarization, and it does not seem to be the best alternative for patients with congenital heart disease (10). Also, as it was shown in case 3, the inappropriate placement of epicardial leads can exacerbate pacemaker-induced ventricular dysfunction. We consider that in any patient who can be taken to endocardial pacemaker implant, it should be performed preferentially over epicardial approach.

### Second challenge: heart disease in pediatric subjects

In congenital heart disease, it is important to know the cardiovascular anatomical variants, particularly in the case of isomerism, and the conduction system of each type of congenital heart disease to which we are facing (7, 12, 22), as well as performing an appropriate electrophysiological study to document the characteristics of the conduction system and the tissues, and thus being able to find the optimal areas on which to implant the electrodes, situation which becomes more important in the case of channelopathies, and where we can face with a structure that is practically inert to electrical stimulation.

Ventricular septal defects can occur in isolation or in association with complex congenital heart disease. The closure of perimembranous interventricular defects is associated with a complete AV block because the conduction system runs in close relation to the posterior edge of the defect (12), and when the defect is closed, the bundle of His can be injured. In the case of attempting physiological stimulation, this can entail a challenge since; although His potential can be evidenced, the block is usually infra-Hisian.

For such situation, stimulation of the left bundle branch is a feasible alternative, where we must not forget the differences in the interventricular septum between children (23) and adults, whose thickness can be more than twice, which should motivate the cautious advancement of the electrode in infants, since the length of the helix of the SelectSecure MRI SureScan 3830 electrode is 1.8 mm, which can allow that with two turns in which the torque is transmitted adequately, interventricular septum perforation is possible.

### Third challenge: how to avoid pacing-induced heart failure in pediatric subjects

All the previously mentioned, together with the fact that cardiac resynchronization through biventricular pacing (CRT-P), have shown less benefit in patients with normal QRS, as well as the fact that left ventricular pacing is performed *via* the epicardial route. In addition, CRT-P has been associated with complications such as phrenic stimulation, venous congestion, mitral valve dysfunction, sepsis, and cerebral vascular events (the last three in the case of the transseptal approach and endocardial placement of the left ventricular electrode) (7, 24). In addition to variations in the position of the coronary sinus, either due to displacement due to heart disease itself or after correction using the different surgical techniques for correcting the defects, this can make it difficult or even impossible the implantation of the electrode for left ventricular stimulation (24). This has aroused interest in the stimulation of the conduction system, either through direct stimulation of the bundle of His or the left bundle branch, since only requiring one electrode simplifies the implantation of the ventricular electrode, not without considering, as already mentioned, the variants in the disposition of the conduction system (7, 12, 22).

Theoretically, His pacing is the ideal resynchronization method. However, the His bundle is only 1–2 mm in diameter, so Hisian pacing remains a challenge (24). In addition to the difficulty in locating His, it has been observed that Hisian pacing has higher pacing thresholds compared to other locations used for cardiac pacing, while left bundle branch pacing has lower capture thresholds both at the time of implantation (left branch 0.6 ± 0.3 V/0.5 ms to 1.1 ± 0.7 V/1 ms vs. His 1.89 ± 1.12 V/0.5 ms to 2.2 ± 1.2 V/1 ms) as chronic thresholds (left branch 1.5 ± 0.6 V/1 ms vs. His 2.4 ± 1.6 V/1 ms) (25, 26).

Physiological stimulation of the conduction system is still in very early stages with some disadvantages already mentioned, such as the Hisian stimulation thresholds, the location of the His potential, as well as the chronic increase and stability of the electrodes. From our point of view, more studies are still required to assess the long-term safety and effectiveness of left septal/left branch stimulation since it does not have the drawbacks of His pacing (see below) and may be much more accessible to the common implanter.

### His bundle pacing limitations

As it has been described by Vijayaraman et al. (10), His bundle pacing has several issues that need to be addressed. The capture thresholds at the His bundle region can be significantly higher than conventional stimulation, because the His bundle is located at the central fibrous body, and, unless the lead penetrates the fibrous insulation or is in proximity, higher energy delivery is needed to be able to capture the His. Acute increase in threshold or loss of capture could be a manifestation of inadequate fixation or displacement of the His bundle lead. The chronic causes are less well understood, and motion of the tricuspid valve may cause unhinging. Local fibrosis and exit block may play a role in chronic thresholds. The increased thresholds cause early battery depletion, that, especially in the pediatric population, implies more device changes during their lifetime, each of which raises the risk of device associated infection. His bundle pacing is time consuming, often requiring long exposition to fluoroscopy. 3D electroanatomical mapping of the His bundle prior to the procedure might help reduce fluoroscopy exposition, at the expense of increased costs for the patient/institution. Finally, the His bundle lead could sense atrial activity. The use of unipolar electrogram can be helpful, since the amplitude is usually better than the bipolar electrograms. Despite that, oversensing and inhibition are still of concern.

## Conclusions

Physiological stimulation could be performed in pediatric patients, with or without structural heart disease. It should be the priority to avoid PM-induced left ventricular dysfunction. The knowledge of anatomical variants of the cardiovascular system and the conduction system in children is essential to decide the ideal approach for this age group.

## Data Availability

The original contributions presented in the study are included in the article/Supplementary Material, further inquiries can be directed to the corresponding author.
