# Supplementary figures and images for: Case report: Challenges and implications of conduction system pacing in pediatrics: Case series
Source: Front Pediatr. 2023 May 4;11:1160335. doi: 10.3389/fped.2023.1160335 (PMC10192706; doi:10.3389/fped.2023.1160335)

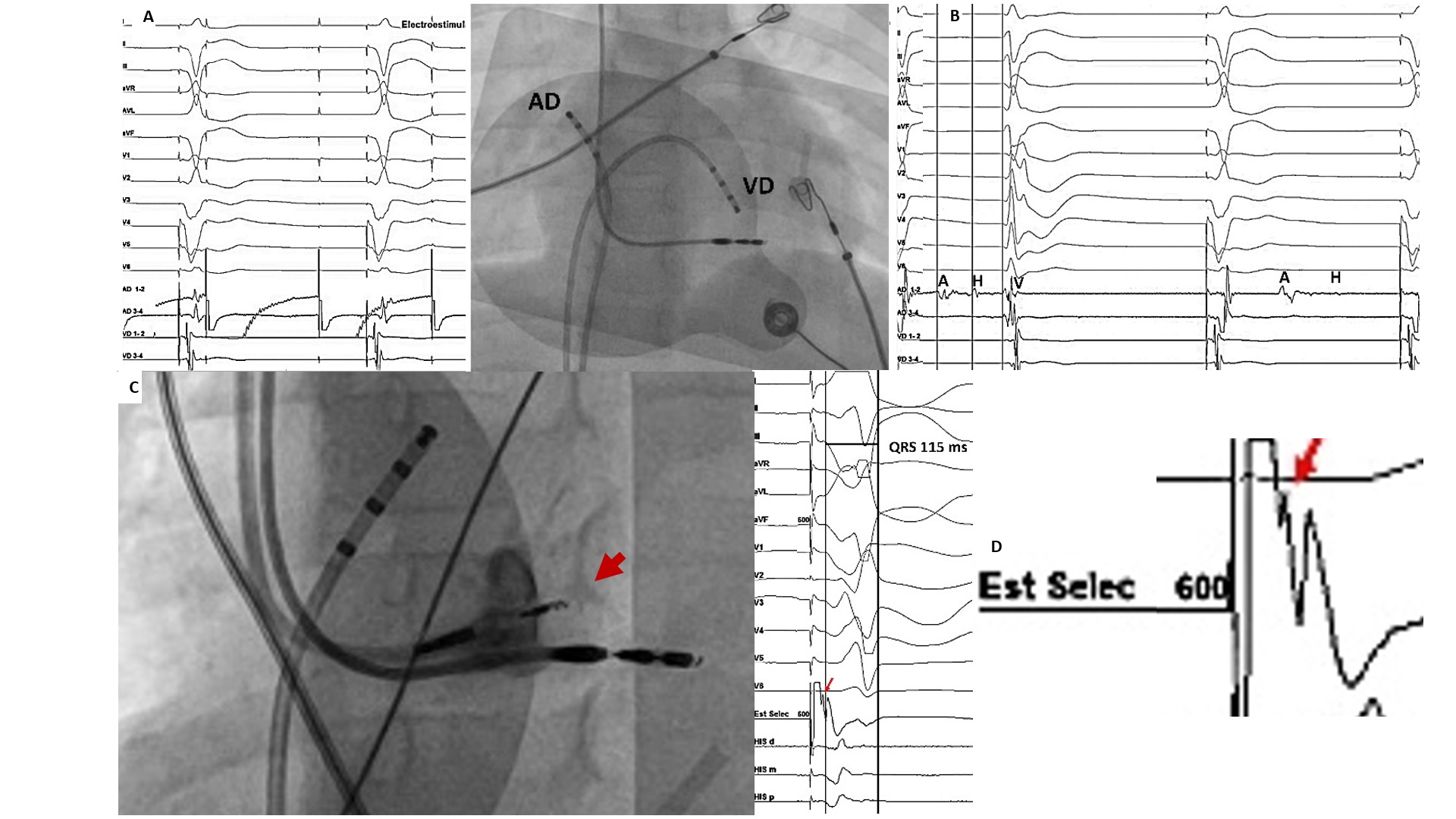

Supplement: Supplementary file 1 [file Image1.jpeg]

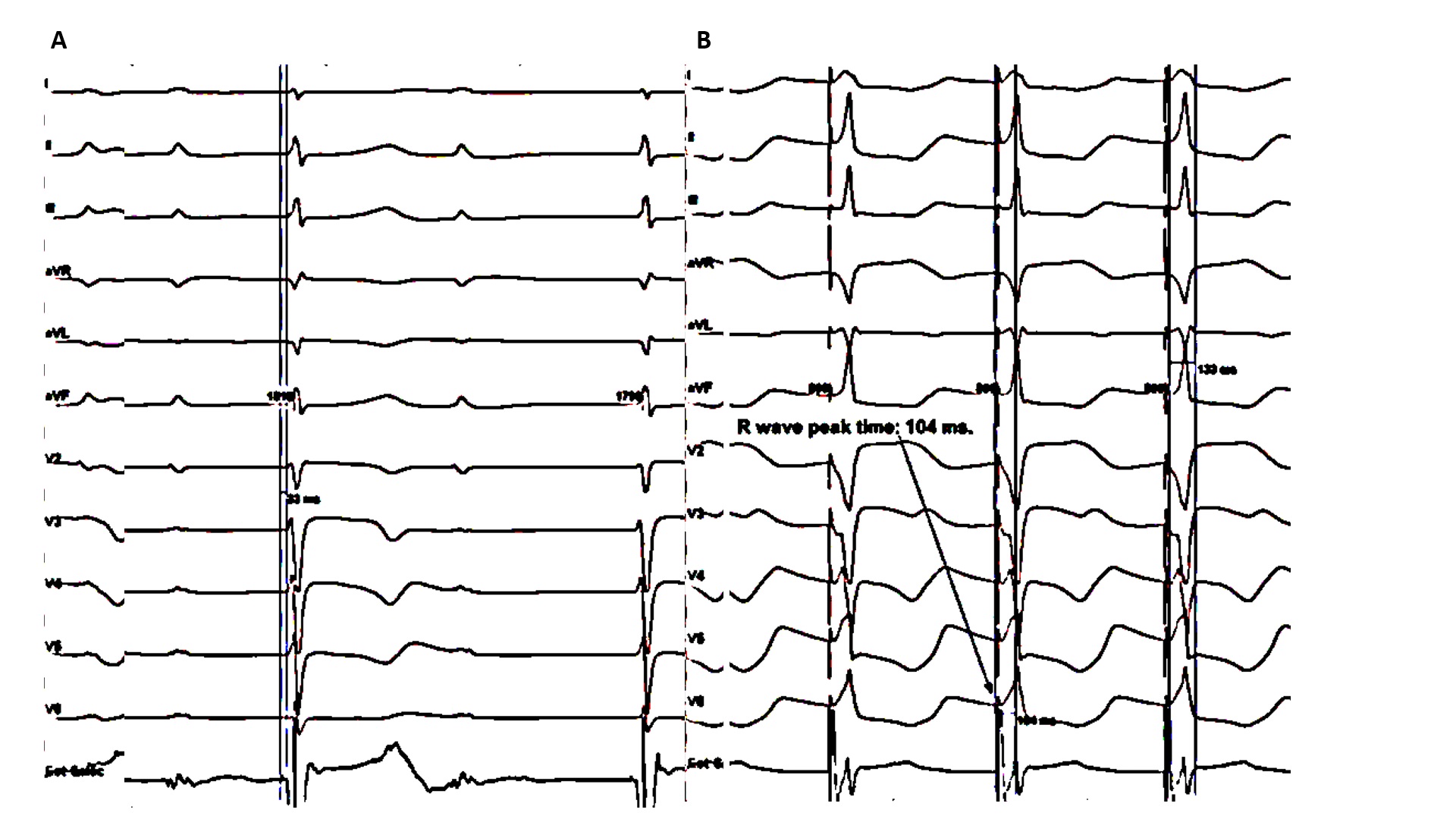

Supplement: Supplementary file 2 [file Image2.jpeg]

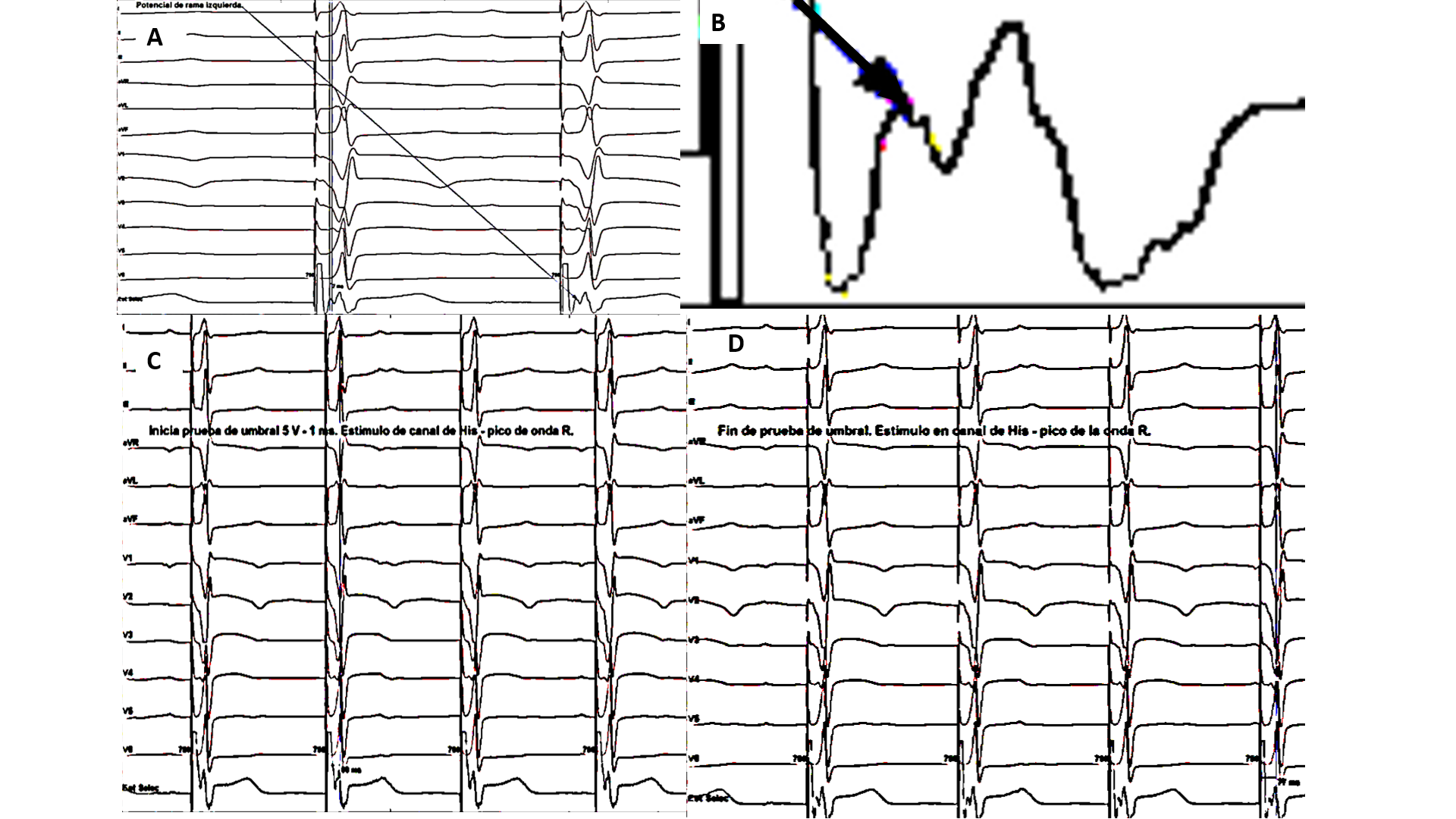

Supplement: Supplementary file 3 [file Image3.jpeg]

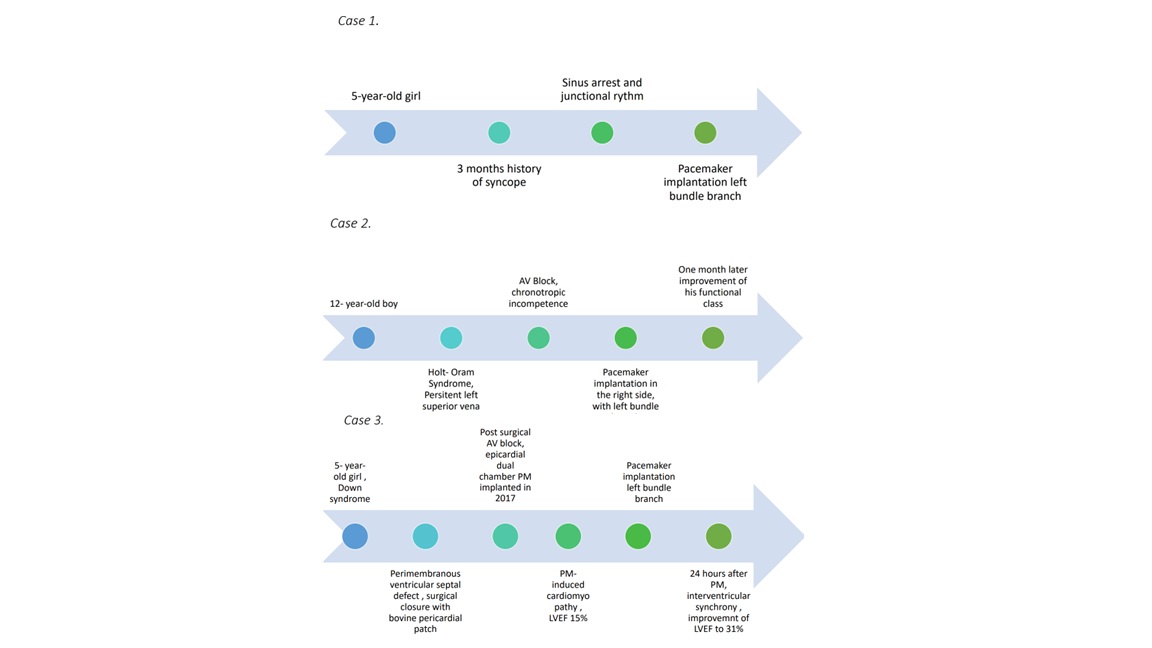

Supplement: Supplementary file 4 [file Image4.jpeg]
